# On the Use of Olive Oil in Large Doses for Softening and Causing the Easy Expulsion of Biliary Calculi

**Published:** 1880-12

**Authors:** 


### Selections.

---

### ON THE USE OF OLIVE OIL IN LARGE DOSES FOR SOFTENING AND CAUSING THE EASY EXPULSION OF BILIARY CALCULI.

THE following paper of Dr. Roderick Kennedy we consider of interest to our readers :

It is scarcely a matter of doubt that the means resorted to for solution and expulsion of biliary calculi have hitherto proved slow and uncertain in their operation. Systematic writers, as a rule, do not attach a great deal of weight to the value of the so-called solvents. Not a few incline to the view that remedies of this kind are practically all but inert. Others assert in plain terms that medicines having these powers do not exist. Chloroform alone, or with ether, in the hands of some, is said to have removed these bodies, but this mode does not seem to have come into general use, probably because it requires time, and the proof of success is inferential. A simple medicine, readily available in practice, and having the power of softening and expelling biliary calculi, it will be admitted, has hitherto been a desideratum. Such a medicine I have, during the past year, used in a variety of cases, and, I am happy to say, always with complete success. In every instance in which the calculi were proved, or presumed, to have been the cause of periodic suffering, these bodies were promptly and painlessly expelled in larger or smaller numbers by the use of large doses of olive oil. In some instances lately, where the patients did not exhibit symptoms of such acute suffering as are more commonly witnessed, but where obstruction to proper flow of bile was evident, I had recourse to this remedy, and in these cases also have been rewarded with similar surprising and satisfactory results.

A brief notice of a few cases illustrating the effects of the means used may prove to be not without interest.

Robert C——, of Adolphustown, an elderly farmer, had for some years been subject to hepatic disorder, attended with the occasional passing of gall-stones. The intervals between the passing of the bodies had gradually become more brief, and lately the paroxysms, always characterized by intense suffering, had come on at intervals of about a week with great regularity. I found the patient anæmic, sallow, and very much exhausted. The usual remedies for the gradual solution of the stones had been tried, but radical relief not being obtained, mitigation of the intense suffering was what, previous to my being sent for, had been principally aimed at. I ordered six ounces of the oil to be taken at bedtime, to be followed in the morning by a full dose of castor-oil. No motion of the bowels was obtained till next evening, after the administration of an enema. Twelve hours after taking the oil, the patient began to complain of a good deal of nausea and faintness, and for several hours there was considerable restlessness, and constant apprehension that the usual paroxysm was about to come on, but no acute pain. In the evening the enema was followed by several copious motions, each containing numbers of softened gall-stones, amounting in all to not fewer than two hundred, varying as to size from a large hickory-nut to a small pea, of a pale yellowish-green color, semi-transparent, soft, easily broken up, and of a great variety of shapes. The patient had a good night's rest, and expressed himself in the morning as feeling very much better, and enjoyed a light breakfast. After the lapse of two days, the paroxysms threatening to return, I ordered the oil to be repeated two nights in succession. The expulsion of a quantity of slimy, bilious-looking matter followed, but no more calculi. The calculi had evidently become dissolved. The patient was left with directions to repeat the use of the oil a few times, at intervals of two or three weeks, and after this as might be indicated by the recurrence of symptoms threatening the return of the paroxysms. I saw the patient five months after my first visit. While there were indications of organic disease of the liver, no more calculi had been passed, and the paroxysms had ceased to return.

Mrs. W. F——, of Ernesttown, presented a case very similar to that of C——. She had for some years suffered from fits of intense pain, generally coming on after a hearty meal. She was directed to take full doses of the oil for two consecutive days. She passed about two hundred calculi, varying in size from a grain of wheat to a filbert. The periodic attacks of pain ceased with the removal of the gall-stones.

Gabriel B—— and Clinton F——, both of this township, each presented a case of hepatic disease, characterized by obstruction to the flow of bile accompanied by severe pain. The oil was administered with the effect of removing softened calculi of considerable size, but in smaller numbers than in the preceding cases, the general symptoms being relieved. A number of other cases might be referred to, in all of which the power of the oil to soften and facilitate the expulsion of biliary calculi was shown to be prompt and unequivocal.

The material point which may be deduced from these cases is that olive oil, administered in repeated large doses, seems to have an unquestionable power of so softening and partially dissolving biliary concretions as to render their expulsion comparatively easy.

Another fact I have noticed is, that although the administration of the oil at intervals of a few weeks or months does not prevent the re-formation of the concretion for the time, yet the resort to the oil alone does not alter the causes or diathesis upon which the formation of these bodies depends.—*The Lancet.*

